# Geography-Driven Evolution of Potato Virus A Revealed by Genetic Diversity Analysis of the Complete Genome

**DOI:** 10.3389/fmicb.2021.738646

**Published:** 2021-10-01

**Authors:** Wei Zhang, Xuhong Sun, Xuyan Wei, Yanling Gao, Jiling Song, Yanju Bai

**Affiliations:** Heilongjiang Academy of Agricultural Sciences, Harbin, China

**Keywords:** potato virus A, genetic diversity, positive selection, phylogeny-trait association analysis, population structure

## Abstract

Potato virus A (PVA), a member of the genus *Potyvirus*, is an important potato pathogen that causes 30%–40% yield reduction to global potato production. Knowledge on the genetic structure and the evolutionary forces shaping the structure of this pathogen is limited but vital in developing effective management strategies. In this study, we investigated the population structure and molecular evolution of PVA by analyzing novel complete genomic sequences from Chinese isolates combined with available sequences from Europe, South America, Oceania, and North America. High nucleotide diversity was discovered among the populations studied. Pairwise *F*_ST_ values between geographical populations of PVA ranged from 0.22 to 0.46, indicating a significant spatial structure for this pathogen. Although purifying selection was detected at the majority of polymorphic sites, significant positive selection was identified in the P1, NIa, and NIb proteins, pointing to adaptive evolution of PVA. Further phylogeny–trait association analysis showed that the clustering of PVA isolates was significantly correlated with geographic regions, suggesting that geography-driven adaptation may be an important determinant of PVA diversification.

## Introduction

Potato (*Solanum tuberosum L.*) is the fourth largest staple crop after rice, wheat, and maize, both worldwide and in China (Qu et al., 2005). Since 1993, China has become the world’s leading potato-producing country (Wang and Zhang, 2004; Jansky et al., 2009), accounting for 26.3% and 22.2% of the global total acreage and yield, respectively (Wang et al., 2011). As a vegetatively propagated crop, potato is prone to infection by more than 50 viruses (Valkonen, 2007; Lesley and Michael, 2020). Among these viruses, six have been recognized as major potato viruses: potato leafroll virus, potato virus Y (PVY), potato virus A (PVA), potato virus M, potato virus X (PVX), and potato virus S (Bai et al., 2007; Zhang et al., 2010; Duan et al., 2018; Mao et al., 2019).

Potato virus A (PVA) has a narrow host range, mainly infecting the members of Solanaceae (Thomas and Nicotiana, 2004). Potato virus A was not officially named until 1932 (Murphy and McKay, 1932), but symptoms suggestive of PVA infection were first reported in 1914 (Orton, 1914). Today, PVA is prevalent in potato production areas worldwide. Normally, the yield loss associated with single PVA infections is moderate, although it can reach 40% in rare cases (Bartels, 1971; German, 2001). However, PVA can infect potato together with many other viruses. In these cases, the yield loss can be much larger (Wang, 1999; Wang et al., 2005; Liu, 2007). For example, double infection of PVA and PVX causes a disease named “potato crinkle” (MacLachlan et al., 1954), which is associated with very severe foliar symptoms and significant yield losses (German, 2001; He et al., 2014; Kreuze et al., 2020). In China, PVA was first discovered on the “Ke shan” variety of potatoes in Heilongjiang Province in 1975. It was subsequently reported in areas including Hunan, Sichuan, Hubei, Zhejiang, Hebei, Fujian, and Guangxi. At present, PVA is prevalent in almost all of the main potato-producing areas in China.

Potato virus A (PVA) causes varying degrees of symptoms, ranging from mild mosaic to severe leaf necrosis, depending on the PVA isolates and potato cultivar (Rajamaki et al., 1998; Nie and Singh, 2001). Relative foliage symptom severity has been used to differentiate PVA isolates into four biological strain groups: very mild, mild, moderately severe, and severe (Bartels, 1971). In addition, Valkonen et al. (1995) and Rajamaki et al. (1998) distinguished four different strain groups (pathotypes) based on whether a PVA isolate caused systemic necrosis (PVA-1), mottle (PVA-2), no infection (PVA-3), or systemic yellowing and stunting (PVA-4) following graft inoculation to the potato cultivar King Edward. A recent study indicated that PVA isolates can be clustered into three monophyletic groups: A, W, and T (Fuentes et al., 2021). Isolates in the A group contain Peruvian potato isolates, whereas the T group comprises three tamarillo isolates from New Zealand. The W group contains isolates with a considerable diversity of sampling locations, and host species (potato and tamarillo). Possibly owing to a fitness advantage over non-recombinants, a substantial increase in the prevalence of A × W recombinant isolates has been observed in South America, Europe and Australia (Fuentes et al., 2021).

As with PVY, the type member of the genus *Potyvirus* (Lefkowitz et al., 2018), PVA has a single-stranded, positive-sense RNA genome ∼10 kb in size. The genome is translated into a large open reading frame (ORF) consisting of a 3,059 amino-acid polyprotein, which is cleaved to yield 10 mature proteins. In addition, a short protein, known as PIPO (Pretty Interesting Potyviridae ORF), was discovered out of frame in the P3 protein (Chung et al., 2008). Studies on the functions of PVA proteins remain limited, although the main functions of the encoded proteins of the genus *Potyvirus* have been systematically summarized (Urcuqui-Inchima et al., 2001; Zhang et al., 2013). Among the proteins encoded in the PVA genome, P1 is a transactive accessory factor during genome amplification and is thought to play an essential role in virus adapting to different host species (Verchot and Carrington, 1995; Valli et al., 2007). NIa is the C-terminus of the endosomal protein NIa. It can perform the catalytic cleavage of polyproteins (Wu et al., 2006). The NIb is an RNA-dependent RNA polymerase (Hong and Hunt, 1996) responsible for viral replication (Li et al., 1997; Wu et al., 2006).

Potato virus A (PVA) is transmitted through infected tubers and mechanical friction, besides being transmitted non-persistently by aphids (Li et al., 1997; Zhang et al., 2010). Potato virus A is one of the oldest potato viruses and has been dated to around 1570CE (Hawkes, 1990; Nunn and Qian, 2010). However, our knowledge on the population genetics and evolutionary biology of PVA is relatively limited compared to other potato pathogens such as *Phytophthora infestans* (Adler et al., 2004; Cardenas et al., 2011) and PVY (Gao et al., 2017; Mao et al., 2019). In this study, we estimated genetic diversity parameters, analyzed population differentiation, identified recombination events, and investigated the role of natural selection during PVA evolution by analyzing the complete genomic sequences of PVA. In addition, we also determined the correlation between the genetic variation and geography of PVA to unveil geography associated adaptation of this virus.

## Materials and Methods

### Virus Isolates

Potato virus A (PVA) isolates were collected from major potato-growing areas in China. Each isolate was maintained a plant of *Nicotiana debneyi* in the lab. The presence of PVA was confirmed by DAS-ELISA (Agdia, Elkhart, United States). Total RNA was extracted from each *N. debneyi* sample using Trizol (Invitrogen, Carlsbad, CA, United States) and reverse-transcribed following the manufacturer’s protocol (Promega, Madison, WI, United States). The complete genome of PVA was obtained by amplifying 10 overlapping fragments (nucleotides 1–409, 200–1428, 1259–2530, 2384–3630, 3476–4730, 4572–5824, 5725–7040, 6889–8160, 7963–9345, and 8986–9567) using 10 pairs of degenerate primers (Supplementary Table 1), which were designed from highly conserved regions of published PVA genomes (accession numbers Nos. KF977085, MT521081, MT435487, MT435489, KM365068, MT502380, and MT502370).

Polymerase chain reaction (PCR) amplifications of cDNA were performed in a total volume of 50.00 μL, containing 2.00 μL of cDNA templates, 25.00 μL of Premix *Taq* (TaKaRa), 1.00 μL of forward primer (10.00 μmol/L), 1.00 μL of reverse primer (10.00 μmol/L), and 21.00 μL of ddH_2_O. The PCR program comprised 5 min at 94°C; 35 cycles of 94°C for 30 s, 48°C–57°C for 30 s (Supplementary Table 1), and 72°C for 1 min; followed by a final extension of 10 min at 72°C. Samples were amplified using a DNA Engine Peltier Thermal Cycler (Bio-Rad Laboratories, Hercules, CA, United States). The PCR products were separated on 1.5% agarose gels in Tris-acetate-EDTA (TAE) buffer and visualized by UV illumination.

PCR products were purified and ligated to pESI-T vector, which was provided in the Hieff Clone ^®^ Zero TOPO-TA Cloning Kit (Yeasen, China), and propagated in cells of *Escherichia coli* strain TOP10. The cloned DNA fragments of recombinant plasmids were sequenced in both directions by Sangon Biological Co. Ltd (Shanghai, China). At least 3–5 independent cDNA clones for each segment were sequenced to assemble consensus sequences.

### Sequence Dataset

Eleven complete or nearly complete genome sequences of PVA isolates, including nine from China, one from Peru, and one from the Netherlands, were obtained in this study and deposited in GenBank under accession numbers MW592838–MW592842 and MW616801–MW616806 (see Supplementary Table 1 for the list of primers used for sequencing). In addition to the novel sequences, 55 complete genome sequences of PVA isolates were downloaded from GenBank (Supplementary Table 2). The sequences had been collected from 14 countries and had known host species and geographic locations. To increase post-analysis interpretability, the isolates were grouped according to their geographic origins. The combined sequence data included China (*n* = 10), Europe (*n* = 15), South America (*n* = 33), Oceania (*n* = 6), and North America (*n* = 2) and were used for the subsequent analyses. Sequences were aligned using the MEGA X (Kumar et al., 2018) and the polyprotein ORF of each sequence was extracted from the alignment. Codon-based sequence alignment was then performed using the MAFFT algorithm (Katoh and Standley, 2013) implemented in PhyloSuite v1.2.2 (Zhang et al., 2020). The program was run using the FFT-NS-I iterative refinement method with the following parameters: mafft –thread 8 –threadtb 5 – threadit 0 –reorder –leavegappyregion. Ambiguously aligned regions were trimmed using the program Gblock 0.91b (Talavera and Castresana, 2007) implemented in PhyloSuite, with the “codon” mode, half gaps allowed, and all other parameters at default settings. The resulting sequence alignment had a length of 9180 nucleotides and used for subsequent population genetics analysis.

### Genetic Diversity and Population Differentiation

To assess how the diversity varied across geographical and host populations, haplotype diversity (*H*_d_) and nucleotide diversity (*P*_i_) were calculated using DnaSP v5.0 (Librado and Rozas, 2009). Analysis of molecular variance (AMOVA) was also carried out using Arlequin v3.5 (Excoffier and Lischer, 2010). The significance of φ-statistics was tested by 1023 random permutations of sequences among the population.

Pairwise among-populations fixation indices (*F*_ST_) were calculated using Arlequin v3.5 (Excoffier and Lischer, 2010), and the significance was obtained with 1000 permutations. A sliding-window analysis was used as an additional approach for evaluating genetic population differentiation. This analysis was performed using the *PopGenome* package (Ver. 2.7.5; Pfeifer et al., 2014) in *R* software (ver. 3.5.1), with a window size of 100 nt and a step size of 30 nt. In addition, discriminant analysis of principal components (DAPC) was used to infer clusters of genetically related individuals. This new multivariate method pioneered by Jombart et al. (2010) was designed to investigate the genetic structure of biological populations without assuming panmixia. In this study, we only performed the DAPC analysis based on pre-defined geographic groups using the *adegenet* package (Ver. 2.0.1; Jombart, 2008) in *R* software (ver. 3.5.1), and therefore, the populations of North America (two potato isolates) and Oceania (three tamarillo isolates) were excluded from the analysis due to an inadequate sample size (*n* ≤ 3).

### Phylogenetic Network and Recombination Analyses

A recent study found evidence for intragenic recombination within the PVA genome (Fuentes et al., 2021). To investigate the role of recombination, we used two different methods to investigate the occurrence of recombination events in CP sequences. A phylogenetic network was first reconstructed using the neighbor-net method implemented in SplitsTree v4.14.8 (Huson, 1998) with default settings. The pairwise homoplasty index (PHI) test implemented in SplitsTree was also carried out to test signals of recombination (*p* < 0.05, significant evidence of recombination). In addition, to confirm the occurrence of recombination in our dataset, localization of recombination breakpoints and identification of likely parental sequences were achieved with the RDP v4.101 package, which incorporates the algorithms RDP, Geneconv, Bootscan, Maxchi, Chimaera, Siscan, and 3Seq (Martin et al., 2015). Recombination events supported by at least four different algorithms of analysis and with *p* values < 1.0 × 10^–5^, viral isolates were identified as recombinants. Because recombinants may result in misleading results in selection analysis, as reported by Anisimova et al. (2003) and Sironi et al. (2015), the recombinants were excluded from subsequent selection analysis.

### Selection Pressure Analysis

To measure the selection pressure in the complete PVA genome, we calculated the ratio (ω) of non-synonymous (*d*N) to synonymous (*d*S) substitutions, as it was done in most adaptive evolution studies, using the CodeML program in the PAML package (Yang, 2007) implemented in EasyCodeML v1.4 (Gao et al., 2019). After all recombinants were removed, our selection analysis was based on 58 polyprotein coding-region sequences. The positive selection models (M2a, M8) and their respective null models (M1a, M7) implemented in the site models were used to conduct the adaptive evolution analysis. Likelihood ratio tests (LRTs) were performed twice to compare the difference in the log-likelihoods between the nested codon-based models against an *x*^2^ distribution with the degree of freedom equal to the differences in the number of parameters between the models (Yang, 1998). When the LRTs yielded significant results, the Bayes empirical Bayes (BEB) method was used to identify the codons that were the most likely to be under positive selection (Yang et al., 2005).

### Phylogeny–Trait Association Analysis

To assess the geographical and host effects on the PVA population, three statistics, the association index (*AI*), parsimony score (*PS*), and maximum monophyletic clade size (*MC*), were calculated from the posterior tree samples using BaTS v2.0 (Parker et al., 2008). For this analysis, phylogenetic uncertainty was used to investigate phylogeny-trait correlations, with 1000 random permutations of tip locations to estimate a null distribution for each statistic. The results that generated a low *AI* index and *PS* and high *MC* scores with *p* < 0.05 suggested a strong phylogeny–trait association.

## Results

### High Genetic Diversity in the Potato Virus A Population

A data set consisting of 66 complete sequences was included in the analysis. After trimming the ambiguously regions from the alignment, we found that all mutations in the PVA genome are substations, with two sequences as exceptions, which had one (accession number: AJ131403) and two codon deletions (accession number: MT502380), respectively. The 66 PVA isolates in this study were composed of 66 haplotypes with an overall haplotype diversity of 1.00 and nucleotide diversity of 0.077 (Table 1). When the viral isolates were categorized according to geographic origin, the highest nucleotide diversity (0.103 ± 0.019) was found in the Oceania population and the lowest (0.012 ± 0.002) was discovered in the Chinese population. When the isolates were grouped according to host origin, higher genetic variation was observed in viral isolates collected from tamarillo compared with those isolated from potato (Table 1), although only four isolates isolated from tamarillo were analyzed in this study. Stepwise diversity analysis indicated that the fragment spanning nucleotide 1-189 and 2791-2950 (Supplementary Figure 1), which corresponds to the coding region for P1 and PIPO, respectively, is the most variable and conserved region on the genome of PVA. Sixty haplotypes were identified in the 66 nucleotide sequences of P1 with an overall haplotype diversity of 0.994 and nucleotide diversity of 0.104 (Supplementary Table 3). In PIPO, 28 haplotypes were identified in the 66 sequences, with an overall haplotype diversity of 0.876 and nucleotide diversity of 0.025 (Supplementary Table 3).

**TABLE 1:** Genetic diversity parameter estimates for PVA population.

Population	Sample size	Haplotype	Haplotype diversity	Nucleotide diversity
**Region**				
China	10	10	1.000 ± 0.045	0.012 ± 0.002
Europe	15	15	1.000 ± 0.024	0.019 ± 0.001
North America	2	n/a	n/a	n/a
Oceania	6	6	1.000 ± 0.096	0.103 ± 0.019
South America	33	33	1.000 ± 0.007	0.086 ± 0.005
**Host**				
Potato	62	62	1.000 ± 0.003	0.069 ± 0.008
Tamarillo	4	4	1.000 ± 0.177	0.088 ± 0.040
Combined	66	66	1.000 ± 0.003	0.077 ± 0.009

Significance thresholds: *, 0.01 < p < 0.05. **, 0.001 < p < 0.01. ***, p < 0.001.n/a, data not available due to an insufficient sample size (n ≤ 3).

### Genetic Differentiation and Population Structure

**FIGURE 1:**
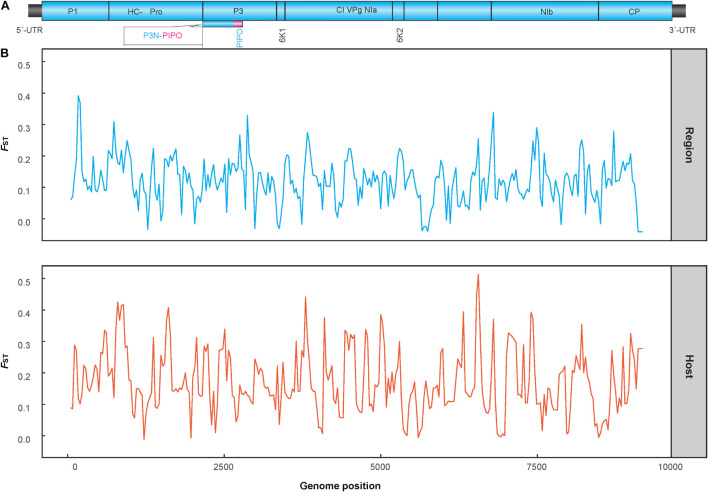
Diagram showing the genomic organization of potato virus A **(A)** and Sliding-window analysis of population differentiation across geographical and host populations **(B)**. *F*_ST_ values were calculated using the R package of *PopGenome*. The window size is 100 nucleotides, and the step size is 30 nucleotides.

The genetic differentiation between all populations of geographic regions was significant, with the *F*_ST_ values ranging from 0.22 to 0.46 (Table 2), indicating significant genetic differentiation between geographic groups of PVA. Similarly, the genetic differentiation between viral isolates with different host species had an *F*_ST_ value of 0.45. The results of sliding-window analysis of the pairwise *F*_S__T_ values of population differentiation among geographic regions and host species are illustrated in Figure 1. The pairwise *F*_ST_ values estimated based on the geographic groupings were similar to those based on the host species groupings. This was in agreement with the pairwise *F*_ST_ analysis above (Table 2).

**TABLE 2:** Pairwise *F*_ST_ between geographic populations of PVA.

	China	Europe	North America	Oceania	South America
China	/				
Europe	0.22 ***	/			
North America	n/a	n/a	/		
Oceania	0.46 ***	0.45 ***	n/a	/	
South America	0.29 **	0.27 **	n/a	0.24 *	/

*n/a, data not available due to an insufficient sample size (n ≤ 3).*

Significance thresholds: *, 0.01 < p < 0.05. **, 0.001 < p < 0.01. ***, p < 0.001. 0.15 < F_ST_ < 0.25, a high degree of differentiation; F_ST_ > 0.25 a substantial degree of differentiation.

An AMOVA analysis also revealed a significant level of genetic differentiation between the PVA sequences originating from either different geographic origins or host species. As shown in Table 3, the variation among geographic regions accounted for 28.07% of the total variation (Φ_ST_ = 0.281, *p* < 0.001), while the variation within regions accounted for 71.93%. When performing the AMOVA only on viral isolates from potato and tamarillo, similar results were obtained; significant variation among groups made up 45.81% of the total variation (Φ_ST_ = 0.458, *p* < 0.01, Table 3), which accounted for nearly 50% of the total genetic variance of PVA. Taken together, it seems that the effect of host species on the genetic variance of PVA is greater than that of geography.

**TABLE 3:** Analysis of molecular variance for the effects of geography and host species.

Grouping factors	Source of variation	d.f.	Sum of squares	Variance components	Percentage of variation	Fixation index
Region	Among groups	4	5912.858	109.053	28.07	Φ_ST_ = 0.281***
	Within groups	61	17047.158	279.461	71.93	
	Total	65	2076.639	388.51423		
Host	Among pop.	1	2366.265	272.049	45.81	Φ_ST_ = 0.458 **
	Within pop.	64	20593.750	321.777	54.19	
	Total	65	22960.015	593.826		

*d.f., degree of freedom.*

Significance thresholds: *, 0.01 < p < 0.05. **, 0.001 < p < 0.01. ***, p < 0.001.

**FIGURE 2:**
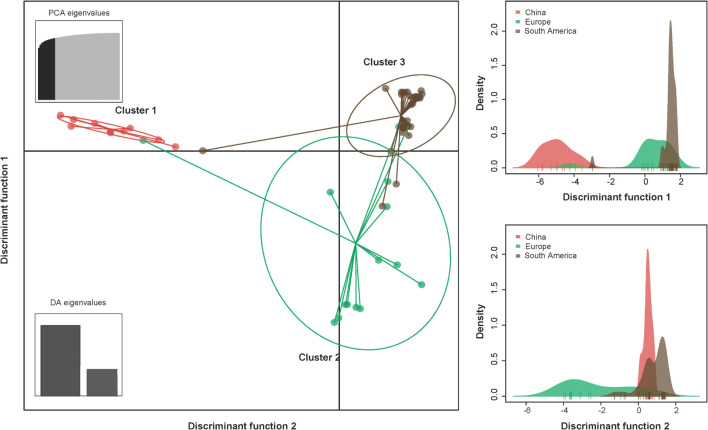
The discriminant analysis of principal components (DAPC) for the geographic structure of PVA isolates from potato. The graph represents the individuals as dots and the groups as inertia ellipses. The bar plots of eigenvalues for the analysis are shown in the inset panel. The density of individuals according to clusters identified along the discriminant function is shown in the right panel. Diagram showing the genomic structure of the PVA genome is shown in the top panel. Viral isolates infecting potato from North America and Oceania were excluded from the analysis due to inadequate sample size (*n* ≤ 3).

The results of the DAPC analysis showed similar patterns of population differentiation as those revealed by the pairwise *F*_ST_ analysis. Discriminant analysis of principal components scatter plots indicated that the population of China were relatively distinct from the other populations along the first discriminant function axis, while the population of Europe exhibited more subtle structure along the second discriminant function axis (Figure 2). Discriminant analysis of principal components scatterplots also showed that the analyzed PVA isolates were divided into three genetic clusters (Figure 2), corresponding to the geographic regions. All the three genetic clusters were clearly differentiated. Cluster 1 contained all PVA isolates from China. Cluster 2 contained 15 individuals from Europe, whereas Cluster 3 comprised 33 individuals from South America.

### Significant Recombination Signals in the Complete Potato Virus A Genome

**FIGURE 3:**
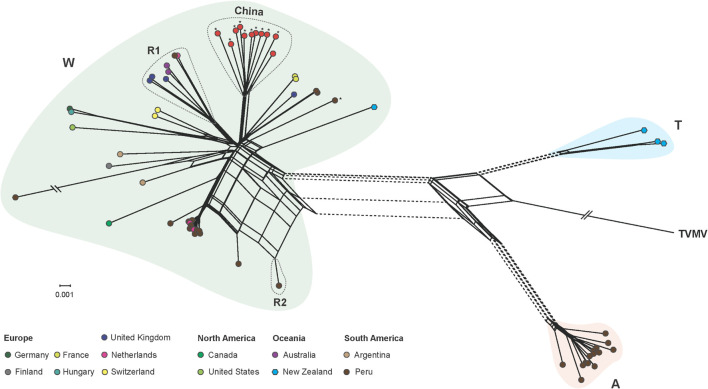
Phylogenetic network inferred from the complete genome of 66 potato virus A isolates. Unique color indicates the geographic origin, as shown in the color key. Phylogenetic groups W (world), A (Andean), and T (tamarillo) were proposed by Fuentes et al. (2021). PVA isolates infecting potato in different regions are indicated by circles, as shown in the color key, and those infecting taramillo are shown in blue hexagons. Isolates sequenced in the study are marked by asterisks. Parallel slashes indicate the branch lengths that were pruned to fit the image size. R1 and R2 indicate recombination group 1 and 2, respectively, which contained recombinants identified by the RDP package. See Table 4 for details of the recombinants. A tobacco vein mottling virus (accession number NC_001768) isolate was used as an outgroup.

Our phylogenetic network analysis showed that PVA isolates were clustered into three lineages (Figure 3), corresponding to the groups W (World), A (Andean), and T (Tamarillo) from the phylogenetic analysis by Fuentes et al. (2021). Three isolates from tamarillo were placed into the T lineage and 14 isolates were placed into the A lineage. The W lineage contained isolates with a considerable diversity of sampling locations. Within the W lineage, all Chinese isolates formed a highly divergent sub-lineage (Figure 3). We also found several conflicting phylogenetic signals that may have been due to recombination (Figure 3), which was supported by the PHI test with statistically significant evidence of recombination (*p* < 0.001). Using the RDP package, 8 PVA isolates were identified as recombinants by at least 4 algorithms (Table 4). In one recombinant (Apu046, accession number MT502353), the breakpoints were detected in the P3 cistron, whereas in all other 7 recombinants (accession numbers GU144321, MT435486, MT435487, MT435489, MT435495, MT502353, MT502377 and MT521083), the breakpoints were detected in the CP cistron (Table 4). The sequences of these eight recombinants were excluded from the selection analysis presented below.

**TABLE 4:** Recombination events detected in the genome of potato virus A by RDP4 Suites.

No	Recombinant	Break point	Parent isolate (Major × minor)	Methods with *p*-value (< 10^–5^)*
1	Apu046	2582-2950	Her × Apu061	G, B, C, S, 3S
2	143-PVA 4631741 20910846 20911289 KIP PE Pun010	8626-9046	CIP706138 × Apu081	B, M, C, 3S

*R, RDP; G, Geneconv; B, BootScan; M, Maxchi; C, Chimarera; S, SiScan; 3S, 3Seq.

### Selection Pressure

**FIGURE 4:**
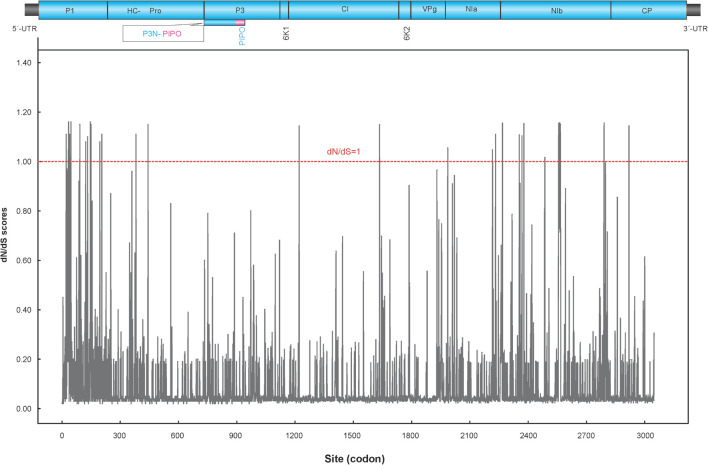
Sliding window plot of *d*N/*d*S ratios across the complete PVA genome. The red dotted line indicates sites under neutral selection (*d*N/*d*S = 1). A diagram showing the genomic structure of the PVA genome is shown in the top panel.

Fifty-eight non-recombinant sequences were included in the selection and phylogeny-trait association analyses. The ratio of mean *d*N/*d*S (less than 1) of the polyprotein coding region showed that the majority of polymorphic sites (98.85%) were under purifying selection (Figure 4), suggesting that most of mutations in the genome were deleterious and consequently being weeded out by natural selection. However, the LRT indicated that the positive selection models (M2a and M8) were significantly better than the control models (M1a and M7), providing evidence for the presence of codons under positive selection. Further analysis from BEB scores indicated a strong positive selection pressure on nine codons, including the P1 (codon sites 34, 46, and 146), NIa (codon site 2268), and NIb cistrons (codon sites 2557, 2560, 2561, 2563, and 2591), with high posterior probability ≥0.95 (Table 5).

**TABLE 5:** Site model tests on the complete genome of PVA.

Model	np	Ln L	Parameter estimates	Compared Models	LRT *p*-value	Positively selected sites
M0	116	–37368.185	ω_0_ = 0.082	M0 *vs.* M3	< 0.001	
M3	120	–36932.676	*p*_0_ = 0.856 *p*_1_ = 0.071, *p*_2_ = 0.073 ω_0_ = 0.023, ω_1_ = 0.2850, ω_2_ = 0.720			Not analyzed
M1a	117	–36959.972	*p*_0_ = 0.9272, *p*_1_ = 0.073 ω_0_ = 0.039, ω_1_ = 1.000	M1a *vs.* M2a	< 0.001	
M2a	119	–36969.264	*p*_0_ = 0.929, *p*_1_ = 0.032, *p*_2_ = 0.039 ω_0_ = 0.039, ω_1_ = 1.000, ω_2_ = 1.000			Not found
M7	274	–5097.661	*p* = 0.127, *q* = 0.848	M7 *vs.* M8	< 0.001	
M8	119	–36904.002	*p*_0_ = 0.967, *p* = 0.110, *q* = 1.040, *p*_1_ = 0.032, ω = 1.156			34*, 46**, 146**, 2268*, 2557*, 2560*, 2561*, 2563*, 2591*

*np, the number of parameters.*

*posterior probability ≥ 0.95; **posterior probability ≥ 0.99.

### Geography-Driven Adaptation of Potato Virus A

With the exception of viral isolates from Europe (*MC* = 2.00, *p* > 0.05), significant signal for geographic clustering was found when PVA isolates were grouped by their sampling regions based on tests of phylogeny-trait association analysis (*MC*: *p* < 0.05, Table 6), indicting a great spatial structure of the pathogen. However, we accepted the null hypothesis of no association between host species and phylogenetic relationships when the PVA isolates were grouped by their host origins (*MC*: *p* > 0.05, Table 6). Taken together, the BaTS results suggested that geographic effects contributed to the diversification of the virus, which could be explained by geography-driven adaptation.

**TABLE 6:** Analysis of the geographic and host effects on the population structure of PVA.

Analyses	Statistic	Observed Mean (95% HPD)	Null Mean (95% HPD)	*p*-value
Region	*AI*	1.50(1.25,1.70)	4.01(3.37,4.68)	< 0.001***
	*PS*	13.23(13.00,14.00)	25.20(23.43,26.94)	< 0.001***
China	*MC*	6.98(7.00,7.00)	1.46(1.00,2.23)	0.01**
Europe	*MC*	2.00(2.00,2.00)	1.47(1.00,2.16)	0.18^ns^
North America	*MC*	n/a	n/a	n/a
Oceania	*MC*	3.00(3.00,3.00)	1.13(1.00,2.00)	0.01**
South America	*MC*	14.00(14.00,14.00)	3.22(2.09,4.56)	0.01**
Host species	*AI*	0.34(0.33,0.34)	0.56(0.15,0.91)	0.100^ns^
	*PS*	3.00(3.00,3.00)	2.96(2.90,3.00)	1.000^ns^
Potato	*MC*	18.34(18.00,19.00)	17.26(8.00,34.07)	0.40^ns^
Tamarillo	*MC*	1.00(1.00,1.00)	1.04(1.00,1.10)	1.00^ns^

AI, association index; PS, parsimony score; MC, maximum monophyletic clade; HPD, highest probability density interval; n/a: No data available due to an insufficient sample size (n ≤ 3). Significance thresholds: *0.01 < p < 0.05; **0.001 < p < 0.01; ***p < 0.001.

## Discussion

In this study, we obtained new sequence data for 11 PVA isolates from China, Peru, and the Netherlands. Combing these data with available sequences retrieved from GenBank, we investigated the genetic diversity and population structure of PVA.

Due to high mutation rates, short generation times, and large population sizes, RNA viruses exhibit extreme evolutionary dynamics (Garcia-Arenal et al., 2001). Consistent with previous studies by Rajamaki et al. (1998) and Kekarainen et al. (1999), a high level of genetic diversity was found for PVA (Table 1) in the current study. This high genetic diversity allows plant RNA viruses, including PVA, to rapidly evolve and adapt to the changing environment (Holmes, 2009).

Recombination plays a major role in shaping genome variation (Gibbs and Ohshima, 2010; Pérez-Losada et al., 2015). Recombinants have also been reported in members of the genus *Potyvirus*, including PVY (Quenouille et al., 2013) and PVA (Fuentes et al., 2021). In this study, similar recombinants were found in the W group proposed by Fuentes et al. (2021). However, no recombinants were identified in PVA isolates from China, which were clustered into a subgroup, showing distinct geographic features (Figure 3). There is one possible explanation for this observation. One is that there is strong selective pressure against the survival of new PVA recombinants of Chinese isolates. Indeed, we found that the large majority of codons in the PVA genome were under purifying selection, suggesting that there are very strong evolutionary constraints acting on PVA and most mutations in the genome were harmful and were subsequently removed by natural selection through reduced survival. However, nine codons in the P1, NIa and NIb proteins were detected to be under positive selection with high confidence levels (posterior probability > 0.95, Table 5). A previous study indicated that most positively selected amino acid sites in the genome of a potyvirus were located to cistrons with hypervariable nucleotides (Wokorach et al., 2020). Consistent with this, the positively selected sites of PVA detected in this study were located to P1, NIa and NIb cistrons. P1, the first protein of the polyprotein, is the most variable protein among potyviruses or within a specific potyviral species (Adams et al., 2005). It is suggested that P1 is involved in adaptation of a potyvirus to a new host species (Valli et al., 2007). Similarly, NIa and NIb also show higher than average genomic variation in PVA (Supplementary Table 3). However, our inferences were drawn solely from the genomic analysis of PVA sequences. Further investigations combining the pathology and biology of this virus will lead to a more comprehensive view of its evolutionary history.

Geographic and host factors were major contributors to the evolutionary dynamics of viruses. The phenomenon is also prevalent in the potyviruses, including PVY (Cuevas et al., 2012), chilli veinal mottle virus (Gao et al., 2016), and Ornithogalum mosaic virus (Gao et al., 2018). Although the phylogenetic network (Figure 3) did not seem to show a clear geography-specific or host species-specific clustering, significant geographical differentiation of PVA was found by more robust AMOVA and sequence-geography association analyses (Tables 3, 6). One explanation for the geographical differentiation is that PVA is a quarantine pest for many countries and agencies. In China, for example, it has been considered a potentially dangerous pest species since 1992. This may have imposed a significant limitation to the international dispersal of PVA.

It should be noted that there are many limitations to this study. For example, the dataset is small and the sequences are very unevenly distributed with respect to their geographical and host origin. Nevertheless, this study represents the first attempt to understand the genetic diversity of PVA at a global level.

## Conclusion

In summary, the present study examined the genetic diversity and population structure of PVA and investigated the role of natural selection during the evolution of PVA. We found that genetic variations were correlated with geographic regions and may have been caused by geographically driven adaptation. In addition, we found evidence of diversifying selection in the genome of this pathogen. These results will be helpful in further studies on the molecular biology of PVA and are essential to understanding the adaptive evolution of this virus.

## Data Availability Statement

The datasets presented in this study can be found in online repositories. The names of the repository/repositories and accession number(s) can be found in the article/Supplementary Material.

## Author Contributions

YB conceived the study. WZ, XS, and XW performed the experiments. WZ, XS, and YB analyzed the data and interpreted the results. WZ and YB led the writing of the manuscript. All authors contributed to the manuscript and agreed on the manuscript before review.

## Conflict of Interest

The authors declare that the research was conducted in the absence of any commercial or financial relationships that could be construed as a potential conflict of interest.

## Publisher’s Note

All claims expressed in this article are solely those of the authors and do not necessarily represent those of their affiliated organizations, or those of the publisher, the editors and the reviewers. Any product that may be evaluated in this article, or claim that may be made by its manufacturer, is not guaranteed or endorsed by the publisher.

## Supplementary Material

Supplementary Table 1 Sequences of degenerate primers used to amplify overlapping segments of potato virus A genome.

Supplementary Table 2 Isolates of potato virus A used in this study.

Supplementary Table 3 Genetic diversity parameter estimates for difference protein coding regions gene in the genome of potato virus A.
