# Supplementary material for: Geography-Driven Evolution of Potato Virus A Revealed by Genetic Diversity Analysis of the Complete Genome
Source: Front Microbiol. 2021 Oct 1;12:738646. doi: 10.3389/fmicb.2021.738646 (PMC8517508; doi:10.3389/fmicb.2021.738646)
Supplement: Supplementary Table 1 — Sequences of degenerate primers used to amplify overlapping segments of potato virus A genome. [file Table_1.DOCX]

**Table S1** The specific primers of the genome of potato virus A

| Amplified fragment | Primer | Sequence (5´–3´) | Position in genome | Product size (bp) |
| --- | --- | --- | --- | --- |
| PVA1 | 1-F | AAAATAAACAAACTACAAAACACAATC | 1-409 | 409 |
|  | 1-R | CAGCTCATGTGGTGCTTACTT |  |  |
| PVA2 | 2-F | CCTGGAGGTTAACTGTAAGCC | 200-1428 | 1229 |
|  | 2-R | AGCCACCAACTATCTTCAATAT |  |  |
| PVA3 | 3-F | TTTCAAAGAGTTCCAGACACA | 1259-2530 | 1272 |
|  | 3-R | TATGGTTGCTCATGTATTATCTC |  |  |
| PVA4 | 4-F | GAGCAACTCACTAGACTCTGAA | 2384-3630 | 1247 |
|  | 4-R | CTAGACTTTGAAACTGAACTGTA |  |  |
| PVA5 | 5-F | GTCTGAGCAGGTTAATTTTGAGCG | 3476-4730 | 1255 |
|  | 5-R | GTGGCCAATTCGTAAAGCAACC |  |  |
| PVA6 | 6-F | GATGCAGTCATAGACTTTGGGA | 4572-5824 | 1253 |
|  | 6-R | TTTGTTTTTCCTTTCTTTGTGTA |  |  |
| PVA7 | 7-F | CCAGAGCCAGAGATGAGAAGAT | 5725-7040 | 1316 |
|  | 7-R | TGCCACTGCCTGGATGTTT |  |  |
| PVA8 | 8-F | GGGGTTCATTCCATCTGC | 6889-8160 | 1272 |
|  | 8-R | ATAAGTCTTTCTTGTCTTTGCTA |  |  |
| PVA9 | 9-F | TGCATTATGCCTTACTTA | 7963-9345 | 1383 |
|  | 9-R | TCGGTTACACCCCCTTCACG |  |  |
| PVA10 | 10-F | ACCATGCAAAGCCTTCTT | 8986-9567 | 582 |
|  | 10-R | CCCTGACAGTTGAAACATAA |  |  |
