# Supplementary material for: Geography-Driven Evolution of Potato Virus A Revealed by Genetic Diversity Analysis of the Complete Genome
Source: Front Microbiol. 2021 Oct 1;12:738646. doi: 10.3389/fmicb.2021.738646 (PMC8517508; doi:10.3389/fmicb.2021.738646)
Supplement: Supplementary Table 2 — Isolates of potato virus A used in this study. [file Table_2.DOCX]

**Table S2** Isolates of potato virus A used in this study

| **No.** | **Isolate** | **Host** | **Country** | **Geographic group** | **Collection date** | **Accession No.** |
| --- | --- | --- | --- | --- | --- | --- |
| A01 | PVA2 | Potato | Canada | North America | 2005 | MH069210 |
| A02 | U | Potato | United States | North America | n/a | AJ131402 |
| A03 | 22 | Potato | France | Europe | 1985 | MT435485 |
| A04 | 622 | Potato | France | Europe | 1985 | MT435493 |
| A05 | 4631723 | Potato | Netherlands | Europe | 2016 | MT521081 |
| A06 | 4631741 | Potato | Netherlands | Europe | 2016 | MT521083 |
| A07 | 5998981 | Potato | Netherlands | Europe | 2016 | MT521082 |
| A09 | 613 | Potato | Switzerland | Europe | 1985 | MT435492 |
| A10 | 775 | Potato | Switzerland | Europe | 1985 | MT435494 |
| A11 | B11 | Potato | Hungary | Europe | 1983 | AJ296311 |
| A12 | Her | Potato | Finland | Europe | n/a | AJ131400 |
| A13 | Ali | Potato | Germany | Europe | 1978 | AJ131401 |
| A14 | 143-PVA | Potato | United Kingdom | Europe | May-08 | GU144321 |
| A15 | GW | Potato | United Kingdom | Europe | 1982 | MT435488 |
| A16 | 20911289 | Potato | United Kingdom | Europe | 2009 | MT435487 |
| A17 | 20910846 | Potato | United Kingdom | Europe | 2009 | MT435486 |
| A18 | PE | Potato | Australia | Oceania | 1992 | MT435495 |
| A19 | KIP | Potato | Australia | Oceania | 2008 | MT435489 |
| A20 | LL6 | Tamarillo | New Zealand | Oceania | 2008 | KM365069 |
| A08 | TamMV | Tamarillo | New Zealand | Oceania | n/a | AJ131403 |
| A21 | B14 | Tamarillo | New Zealand | Oceania | 2008 | KM365068 |
| A22 | 7 | Tamarillo | New Zealand | Oceania | 1989 | KM365067 |
| A23 | CIP710179 | Potato | Argentina | South America | 1987 | MT502382 |
| A24 | CIP705869 | Potato | Argentina | South America | 1987 | MT502381 |
| A25 | CIP706138 | Potato | Peru | South America | 1986 | MT502380 |
| A26 | CIP704104 | Potato | Peru | South America | 1974 | MT502379 |
| A27 | CIP703867 | Potato | Peru | South America | 17-Jun-74 | MT502378 |
| A28 | Pun010 | Potato | Peru | South America | 2-Apr-18 | MT502377 |
| A29 | Hco037 | Potato | Peru | South America | 9-Mar-16 | MT502376 |
| A30 | Hco004B | Potato | Peru | South America | 7-Mar-16 | MT502375 |
| A31 | Hco003B | Potato | Peru | South America | 7-Mar-16 | MT502374 |
| A32 | Cus080 | Potato | Peru | South America | 29-Nov-16 | MT502373 |
| A33 | Cus079 | Potato | Peru | South America | 29-Nov-16 | MT502372 |
| A34 | Apu090A | Potato | Peru | South America | 6-Apr-18 | MT502371 |
| A35 | Apu090 | Potato | Peru | South America | 6-Apr-18 | MT502370 |
| A36 | Apu087 | Potato | Peru | South America | 6-Apr-18 | MT502369 |
| A37 | Apu084 | Potato | Peru | South America | 6-Apr-18 | MT502368 |
| A38 | Apu082 | Potato | Peru | South America | 6-Apr-18 | MT502367 |
| A39 | Apu081 | Potato | Peru | South America | 6-Apr-18 | MT502366 |
| A40 | Apu078 | Potato | Peru | South America | 5-Apr-18 | MT502365 |
| A41 | Apu077 | Potato | Peru | South America | 5-Apr-18 | MT502364 |
| A42 | Apu076 | Potato | Peru | South America | 5-Apr-18 | MT502363 |
| A43 | Apu074 | Potato | Peru | South America | 5-Apr-18 | MT502362 |
| A44 | Apu070A | Potato | Peru | South America | 5-Apr-18 | MT502361 |
| A45 | Apu070 | Potato | Peru | South America | 5-Apr-18 | MT502360 |
| A46 | Apu066 | Potato | Peru | South America | 5-Apr-18 | MT502359 |
| A47 | Apu064 | Potato | Peru | South America | 5-Apr-18 | MT502358 |
| A48 | Apu063 | Potato | Peru | South America | 5-Apr-18 | MT502357 |
| A49 | Apu061 | Potato | Peru | South America | 5-Apr-18 | MT502356 |
| A50 | Apu048 | Potato | Peru | South America | 5-Apr-18 | MT502355 |
| A51 | Apu047 | Potato | Peru | South America | 5-Apr-18 | MT502354 |
| A52 | Apu046 | Potato | Peru | South America | 5-Apr-18 | MT502353 |
| A53 | Apu007 | Potato | Peru | South America | 4-Apr-18 | MT502352 |
| A54 | Apu003 | Potato | Peru | South America | 4-Apr-18 | MT502351 |
| A55 | Hunan | Potato | China | Asia | 30-Apr-10 | KF977085 |
| **A56** | **2_1** | **Potato** | **China** | **Asia** | **2006** | **MW592838** |
| **A57** | **2014_c_356** | **Potato** | **China** | **Asia** | **23-May-14** | **MW616801** |
| **A58** | **2014_c_335** | **Potato** | **China** | **Asia** | **6-Jun-14** | **MW616802** |
| **A59** | **2014_c_328** | **Potato** | **China** | **Asia** | **3-Jun-14** | **MW616803** |
| **A60** | **2012_c_291** | **Potato** | **China** | **Asia** | **8-Jul-12** | **MW616804** |
| **A61** | **2018_15** | **Potato** | **China** | **Asia** | **2018** | **MW592839** |
| **A62** | **2018_8** | **Potato** | **China** | **Asia** | **2018** | **MW592840** |
| **A63** | **KB116** | **Potato** | **Peru** | **South America** | **1990** | **MW616805** |
| **A64** | **KB117** | **Potato** | **China** | **Asia** | **1986** | **MW616806** |
| **A65** | **KB118** | **Potato** | **Netherlands** | **Europe** | **1990** | **MW592841** |
| **A66** | **ST6** | **Potato** | **China** | **Asia** | **2019** | **MW592842** |

Isolates sequences in this study are shown in bold font.

n/a, not data available.
