# Supplementary material for: Geography-Driven Evolution of Potato Virus A Revealed by Genetic Diversity Analysis of the Complete Genome
Source: Front Microbiol. 2021 Oct 1;12:738646. doi: 10.3389/fmicb.2021.738646 (PMC8517508; doi:10.3389/fmicb.2021.738646)
Supplement: Supplementary Table 3 — Genetic diversity parameter estimates for difference protein coding regions gene in the genome of potato virus A. [file Table_3.DOCX]

**Table S3** Genetic diversity parameter estimates for difference protein coding regions gene in the genome of potato virus A

| **Gene** | **Haplotype** | **Haplotype diversity** | **Nucleotide diversity** |
| --- | --- | --- | --- |
| Polyprotein | 66 | 1.000±0.003 | 0.077±0.009 |
| P1 | 60 | 0.994±0.005 | 0.104±0.013 |
| HC-Pro | 66 | 1.000±0.003 | 0.077±0.008 |
| P3 | 58 | 0.989±0.008 | 0.062±0.007 |
| 6K1 | 34 | 0.942±0.016 | 0.077±0.009 |
| CI | 64 | 0.999±0.003 | 0.077±0.008 |
| 6K2 | 34 | 0.955±0.013 | 0.092±0.010 |
| VPg | 58 | 0.989±0.008 | 0.069±0.008 |
| NIa | 56 | 0.988±0.008 | 0.075±0.009 |
| NIb | 63 | 0.998±0.003 | 0.084±0.009 |
| CP | 66 | 1.000±0.003 | 0.077±0.009 |
| PIPO | 28 | 0.876±0.031 | 0.025±0.003 |
