# Supplementary figures and images for: Geography-Driven Evolution of Potato Virus A Revealed by Genetic Diversity Analysis of the Complete Genome
Source: Front Microbiol. 2021 Oct 1;12:738646. doi: 10.3389/fmicb.2021.738646 (PMC8517508; doi:10.3389/fmicb.2021.738646)

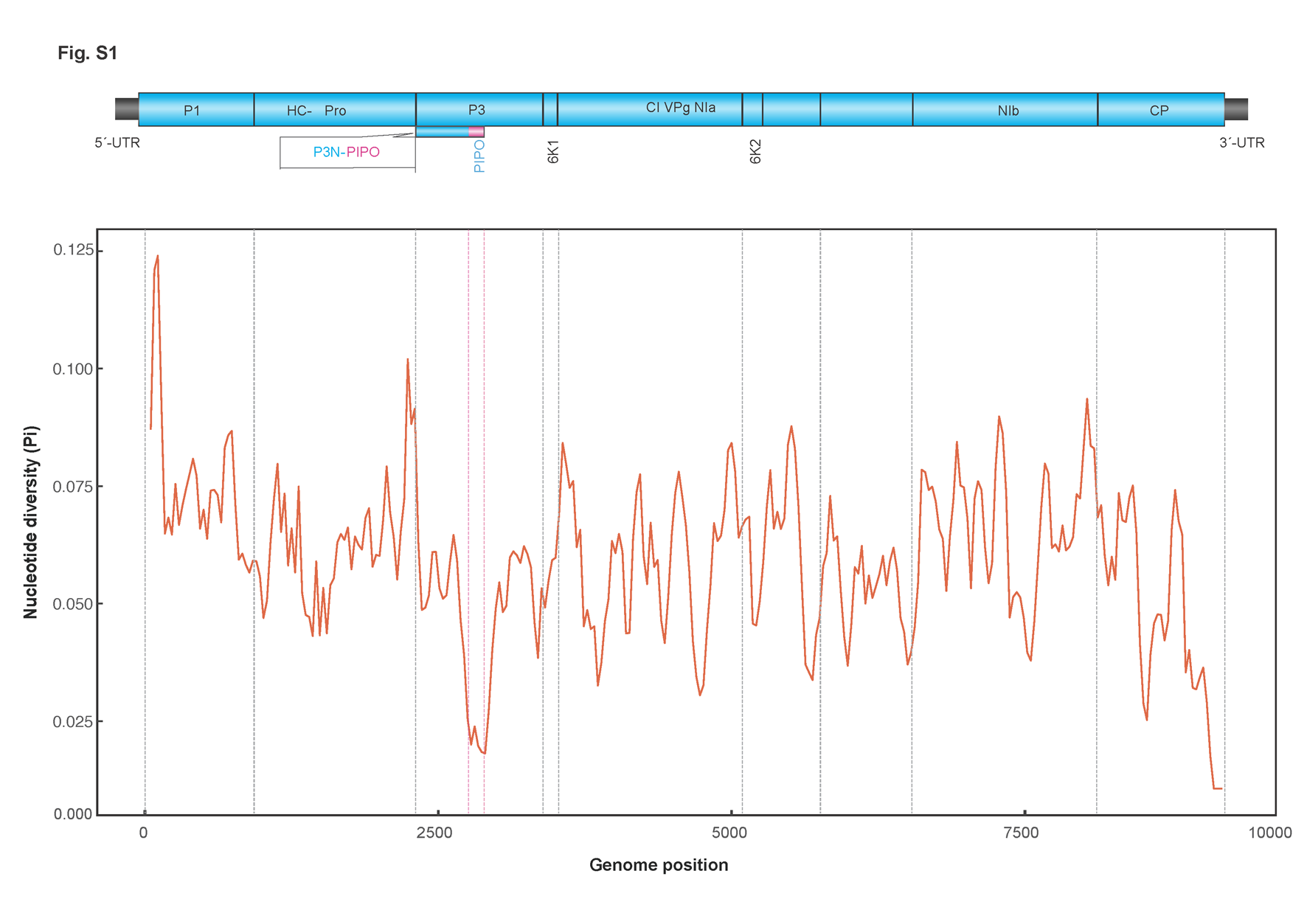

Supplement: Supplementary file 4 [file Image_1.TIFF]
